# Does the Host Contribute to Modulation of Mycotoxin Production by Fruit Pathogens?

**DOI:** 10.3390/toxins9090280

**Published:** 2017-09-12

**Authors:** Dilip Kumar, Shiri Barad, Edward Sionov, Nancy P. Keller, Dov B. Prusky

**Affiliations:** 1 Department of Postharvest Science of Fresh Produce, The Volcani Center, Bet Dagan 50250, Israel; dilip.hcu@gmail.com (D.K.); barad.shiri@gmail.com (S.B.); 2 Department of Food Quality and Safety Agricultural Research Organization, The Volcani Center, Bet Dagan 50250, Israel; edwardsio@volcani.agri.gov.il; 3 Department of Medical Microbiology and Immunology, University of Wisconsin–Madison, 1550 Linden Dr., Madison, WI 53706-1598, USA; npkeller@wisc.edu; 4 Department of Bacteriology, University of Wisconsin–Madison, 1550 Linden Dr., Madison, WI 53706-1598, USA

**Keywords:** mycotoxin, fruit pathogen, food safety, fungal pathogenicity, fruit ripening

## Abstract

Storage of freshly harvested fruit is a key factor in modulating their supply for several months after harvest; however, their quality can be reduced by pathogen attack. Fruit pathogens may infect their host through damaged surfaces, such as mechanical injuries occurring during growing, harvesting, and packing, leading to increased colonization as the fruit ripens. Of particular concern are fungal pathogens that not only macerate the host tissue but also secrete significant amounts of mycotoxins. Many studies have described the importance of physiological factors, including stage of fruit development, biochemical factors (ripening, C and N content), and environmental factors (humidity, temperature, water deficit) on the occurrence of mycotoxins. However, those factors usually show a correlative effect on fungal growth and mycotoxin accumulation. Recent reports have suggested that host factors can induce fungal metabolism, leading to the synthesis and accumulation of mycotoxins. This review describes the new vision of host-factor impact on the regulation of mycotoxin biosynthetic gene clusters underlying the complex regulation of mycotoxin accumulation in ripening fruit.

## 1. Introduction

Fruit spoilage by fungi causes not only substantial economic losses but also health risks upon mycotoxin consumption. Mycotoxins are toxic low-molecular-weight secondary metabolites produced mainly by filamentous fungi belonging to the genera *Aspergillus*, *Penicillium*, *Alternaria*, and *Fusarium*. Mycotoxins, which are secreted with only minor effects on fungal growth onto various foods and feeds, can cause diseases collectively known as mycotoxicoses in humans and animals upon ingestion [1]. The consumed metabolites can affect single or multiple target organs, leading to cytogenic, mutagenic, carcinogenic, teratogenic, or immunosuppressive effects [2]. Hence, in many countries, mycotoxin levels in foods are regulated, and the U.S. Food and Drug Administration limits mycotoxin levels in fruit products [3,4]. This review analyzes the recently discovered factors modulating the occurrence and regulation of mycotoxins in postharvest fruit and provides examples of how the fruit host might modulate the induction of mycotoxin production.

Fruits may become infected with mycotoxigenic fungi through damaged surfaces—mechanical injuries, insect wounds, cuts, and splits—that occur during growing, harvesting, packing, transporting, postharvest storage, and marketing. Toxin production is dependent on a variety of complex interactions between internal and environmental factors, including the geographical location where the fruit is grown and harvested, the humidity, temperature, pathogen load on the fruit, fungal strain, fruit type and cultivar, ripening, and fruit physiological properties [5,6]. Fruit maturity at harvest is believed to be an important factor in susceptibility to infection by pathogenic fungi during postharvest storage, due to high sugar content, water activity (a_w_), changes in pH, decreased firmness, and weakening defense systems [7,8,9].

There are large numbers of known mycotoxins but only a few are commonly found in fruit: aflatoxins in dried figs (*Aspergillus flavus*), ochratoxin A in grapes, peaches, cherries, strawberries, apples, and dried figs (*Penicillium* and *Aspergillus* species), patulin in deciduous fruits (*Penicillium* and *Aspergillus* species), alternariol in tomatoes, apples, tangerines, and mandarins (*Alternaria* species), citrinin in deciduous fruits (*Penicillium* species) and trichothecenes in sweet pepper (*Fusarium* and *Trichothecium* species) [6,10,11,12]. However, few studies have examined the importance of fruit metabolic changes during ripening in mycotoxin production. Since the infection by mycotoxigenic fungi in fruit occurs in the field, during harvesting, postharvest, and during storage, the physiological changes inducing ripening occurring in the host after infection and their contribution to mycotoxin accumulation are likely of great importance.

## 2. Mycotoxins Commonly Found in Fruit

### 2.1. Patulin

Patulin is a low-molecular-weight α, β-unsaturated γ-lactone found in fruit. This toxin causes severe toxicosis due to neurotoxic, nephrotoxic, mutagenic, teratogenic, or hepatotoxic effects, which can result in nausea, vomiting, kidney damage, and gastrointestinal disorders [13,14,15]. *Penicillium expansum*, an airborne fungus, is the major species contributing to patulin accumulation in apple products [16]. Several other fungi can produce patulin, including *Aspergillus clavatus*, *A. giganteus*, *A. terreus*, *Byssochlamys fulva*, *B. nivea*, and several *Penicillium* species [17,18,19,20]. Patulin can be found in several types of mold-colonized fruit, including pears, plums, berries, and tomatoes, but the major source of patulin contamination is apples and its juice products [21,22,23].

#### Factors Affecting Patulin Production

Various factors are known to affect patulin production in apple, including cultivar type, geographical location, climate, mechanical injury, storage conditions, and pre- and post-harvest conditions.

*Fruit development conditions—*As it matures, the fruit undergoes physiological changes, such as increases in pH and total soluble solids (TSS), decreases in firmness and acidity, and weakening of the defense system, which can increase its susceptibility to pathogen and patulin production [8,9]. Moreover, patulin accumulation in apples is affected by environmental conditions, fungicide residues, pesticide treatments, microbial load, harvest method, and postharvest treatments [24]. Foliar spray of chemicals on apple trees during fruit development results in significant reductions in *P. expansum* infection and consequently, in mycotoxin accumulation [25,26,27].

*Fruit cultivar—*Several reports concluded that fruit cultivars differ in their susceptibility to pathogen attack and patulin accumulation in apples [28,29,30,31,32,33]. Fruit cultivars differ in their physical and chemical properties, such as skin thickness, pH, TSS, acidity, firmness, and defense systems, which may affect patulin production [29,32,34]. A study of four apple varieties showed that patulin accumulation is significantly higher in ‘Red Delicious’ and ‘Golden Delicious’ than in ‘Granny Smith’ and ‘Fuji’, and is negatively correlated with the acidity of the fruit [35,36,37]. In other studies with ‘Red Delicious’, ‘Golden Supreme’, ‘Gala’, ‘Fuji’, ‘Empire’, and ‘McIntosh’, the varieties that showed the highest patulin accumulation were ‘Golden Supreme’ (54.2 μg kg^−1^) and ‘McIntosh’ (52.1 µg kg^−1^) [38]. More recent indications by Snini et al. [39] suggest that patulin is not indispensable for the initiation of the disease, but it acted as a cultivar-dependent aggressiveness factor for *P. expansum* in 13 different cultivars tested. This conclusion was strengthened by the fact that the addition of patulin to apples infected by the PeΔ*patL* mutant lacking one of the genes of the patulin production in the cluster required for patulin synthesis restored normal *P. expansum* colonization in the apples [39].

When colonization of *P. expansum* was compared in pears and apples, its growth was higher in the former; however, the latter tended to accumulate more patulin [32], suggesting that internal compounds may contribute to differential patulin accumulation. The effect of pH was also evaluated in apples: patulin production increased from pH 2.5 to 3.5 and then remained constant to pH 5.5. Other factors, such as fruit organic acid content and degree of ripeness, may play important roles in patulin accumulation [40,41].

*Storage temperature—*Optimal patulin production has been reported in the temperature range of 23–25 °C [30,32]. As the temperature decreased during storage (0–4 °C), patulin production declined but was not completely inhibited [29,30,40,42].

*Environmental factors—*Zong and co-workers [43] reported that environmental factors such as pH, and chemical factors such as carbon (C) and nitrogen (N) sources have large effects on patulin production in *P. expansum* strains. They found glutamic acid to be the best N source and sucrose, maltose, and glucose the best C sources in the promotion of patulin production, and the optimal pH was 5 in different *P. expansum* strains.

### 2.2. Ochratoxin

Ochratoxins are a group of pentaketide mycotoxins found in fruit that are produced mainly by *Aspergillus* and *Penicillium* species [44,45]. The most important and most toxic ochratoxin found naturally in foods is ochratoxin A (OTA), which shows hepatotoxicity, teratogenicity, carcinogenicity, cytotoxicity, neurotoxicity, and immunosuppressive properties [46,47,48,49,50,51]. Two other forms of ochratoxin—B and C—are less toxic and less common [52,53]. OTA is generally produced by *Aspergillus ochraceus* [54] and *Aspergillus carbonarius*, which typically infect wine grapes, and *Aspergillus alliaceus*, which contaminates nuts and figs [55,56].

#### Factors Affecting OTA Production

*Temperature and a_w_*—These factors are likely to affect the rate of growth and OTA production in *Aspergillus* species in grapes. The optimal temperature for OTA production is in the reported range of 25–30 °C for *A. carbonarius*, and 30–37 °C for *A. niger* and *A. ochraceus* [57,58,59,60]. The optimal a_w_ for toxin production is 0.95–0.99 for *A. carbonarius* and 0.90–0.95 for *A. niger* [61]. OTA production is also strongly influenced by culture pH, with large amounts of OTA being produced at pH < 7.0 and reduced amounts at higher pH values in *A. ochraceus* [62,63].

*Fruit development conditions*—OTA accumulates to high levels during *Aspergillus* colonization of grapes with high sugar content (16–20% TSS). When grapes’ sugar content increases, they are more susceptible to infection by *A. carbonarius* and are also capable of supporting OTA production [64,65,66]. Delayed harvest of mature berries also increases the risk of OTA contamination [66,67]. Hot weather coupled with increased humidity and rainfall increased *Aspergillus* incidence and OTA contamination in some studies [68,69]. OTA production was correlated with severity of infection for certain varieties of grapes but not others. Nevertheless, differences in OTA contamination among varieties were often associated more strongly with seasonal variations in climate and time of ripening than with inherent characteristics of the variety [64,70].

*Storage conditions—*OTA is concentrated in berries displaying visible disease damage [65,71]. Cold storage of table grapes (0 °C) with sulfur dioxide-generating pads reduced the incidence of black *Aspergillus* species [72]. In addition, rapid drying of grapes above 30 °C to a safe a_w_ reduced the potential for OTA production [73].

*Nutritional factors—*The type of C source also influences OTA accumulation: glucose, sucrose, maltose, galactose, xylose and glycerol repressed OTA production, whereas the presence of lactose resulted in a ca. 7-fold increase in OTA production by *A. ochraceus* [56]. Addition of other C sources, such as arabinose, to fungal media increased OTA accumulation by *A. ochraceus*, *A. carbonarius*, and *A. tubingensis* [74]. N sources, specifically ammonium nitrate and acetate, also enhanced OTA production [75] compared to ammonium sulfate and chloride [56,63]. Abbas et al. [56] also indicated that other organic N sources, such as urea and glutamine, enhance OTA synthesis by *A. ochraceus*, and phenylalanine specifically favored OTA production in *A. ochraceus*, *A. carbonarius*, and *A. tubingensis* [74].

In a recent work, systematic expression analysis of OTA mycotoxin biosynthesis genes was performed to examine the relationship between growth and general expression patterns in relation to single environmental factors, such as temperature, a_w_, and pH, and a_w_–temperature interactions [76]. Abiotic factors such as temperature, a_w_ and pH were found to have a strong influence on the expression of mycotoxin biosynthesis genes, in agreement with the findings of several other studies [77,78,79,80]. The expression profile pattern was more pronounced in relation to changes in temperature and a_w_ than to changes in pH.

### 2.3. Alternaria Toxins

*Alternaria* species are saprophytic in nature, and widely distributed in the soil and on plant surfaces. Several species are known to grow well at low temperatures and are responsible for fruit spoilage during refrigerated transport and postharvest storage, causing severe postharvest economic losses [23,81]. *Alternaria* species have been reported to produce mycotoxins in fruit [82,83,84,85] such as apples, berries, oranges, tomatoes, lemons, and grapes [81,86,87]. *Alternaria* species are also common pathogens of tomatoes, peppers, and eggplant, in which fungal infection is generally initiated by wounds at the calyx scar [88,89]. The most important *Alternaria* mycotoxins can be grouped into three different structural classes: alternariol (AOH) and its monomethyl ether (AME), as well as altenuene, all dibenzopyrone derivatives; tenuazonic acid (TeA), a tetramic acid derivative, and altertoxins I, II, and III, which are perylene derivatives. The possibility that *A. alternata* may be a factor in several types of cancer was confirmed in several studies [90], where it was indicated that those toxins may cause cell mutagenicity and in combination with human fetal esophageal epithelium DNA, activate oncogenes [90].

#### Factors Affecting *Alternaria* Toxins

*Alternaria* mycotoxin production in host fruit depends upon fungal strain, host species, cultivar, and fruit maturity.

*Fruit development and wounding conditions*—Temperature is one of the important factors affecting the rate of colonization of *Alternaria*. Apples can be infected by *Alternaria alternata* through wounds occurring during fruit development, harvesting and storage, leading to tissue colonization and toxin accumulation. Mild temperatures (10–25 °C), relative humidity higher than 80%, and tissue susceptibility are the most strongly determinant factors for infection [6,91]. For example, *Alternaria* brown spot disease in citrus and persimmon fruit is more severe during conditions of rainfall and high relative humidity [92,93]. In tomatoes, host nutritional deficiencies and skin burn, together with warm and rainy weather, also enhance *Alternaria* infection. In naturally infected tomato fruit in Italy, the *Alternaria* mycotoxin TeA may reach up to 7200 µg kg^−1^ [94].

*Storage conditions—*Ozcelik et al. [95] reported the inhibitory effects of high-density polyethylene film packaging on *Alternaria alternata* colonization compared to on unpackaged tomatoes, showing a simultaneous reduction in AOH and AME and partial inhibition of fungal growth. These results indicated that differential levels of CO_2_ and O_2_, which affect fungal development, may modulate mycotoxin production. Toxin production in synthetic media by *Alternaria alternata* was optimal (depending upon the type of toxin—AOH, AME, or TeA) in a temperature range of 14–28 °C [96,97]. Pose et al. [97] suggested that the most favorable temperature for AOH synthesis is 21 °C over the a_w_ range of ca. 0.922–0.982, whereas maximum AME production was at a_w_ = 0.954 and 35 °C. This could explain Hasan’s [96] finding that postharvest storage of tomato fruit at low temperature (7 °C) reduces fungal growth and toxin production.

## 3. Fungal and Host Regulation of Mycotoxin Synthesis in Fruit

### 3.1. Environmental Effects on Gene-Biosynthesis Pathways of the Fungus

As we have seen, the biosynthesis of mycotoxins is highly regulated, and substrate [98], temperature [57,99], a_w_ [100], and pH [101] can have profound effects. However, aside from the wide description of factors that have been reported to modulate mycotoxin production, few reports have shown the effects of environmental profiles on optimal gene activation and concurrent mycotoxin synthesis. This has been determined for OTA biosynthesis by *Penicillium verrucosum* [101,102,103], trichothecene biosynthesis by *Fusarium* [104,105,106], and aflatoxin biosynthesis by *Aspergillus* [107,108]. The recent systematic investigation of these parameters’ influence on the expression of mycotoxin biosynthesis genes [62,76,78,80,109,110] has clarified the mechanism governing external factors’ influence on the transcript expression of genes for mycotoxin biosynthesis [76]. Several other factors, such as the positive influence of oxygenic stress described on aflatoxin biosynthesis by *Aspergillus parasiticus* [111] or the application of suboptimal concentrations of some fungicides, e.g., strobilurins, can have a strong effect on mycotoxin biosynthesis [112]. Furthermore, for *P. verrucosum*, suboptimal concentrations of preservatives, such as calcium propionate and potassium sorbate, have been shown to lead to an increase in OTA biosynthesis. In parallel, it was shown that the *otapksPV* gene (encoding OTA polyketide synthase), a key gene in the OTA biosynthesis pathway, is activated under these stress conditions. This demonstrated, for the first time, that environmental factors modulate gene biosynthesis pathways [110]. Stress activation was also demonstrated for the *fum1* gene of *Fusarium verticillioides* [80].

#### Host Effects on the Fungus

Fruits are fresh products that undergo significant biochemical and chemical changes during postharvest storage. Fruit nutritional components that undergo changes during fruit maturation and ripening include sugars (levels may increase from 5 to 20%) and organic acids, coupled with an increase in fruit pH [79]. This raises the question of whether such changes in the host can modulate the expression of fungal mycotoxin biosynthetic gene clusters (BGCs).

With respect to host-produced metabolites, Barkai-Golan, Paster, Jackson, and Al-Taher [113,114] claimed that the high content of carbohydrates in figs, dates, citrus fruit, and raisins probably enhances aflatoxin production by *Aspergillus flavus* and *Aspergillus parasiticus*; however, no modulatory mechanism for cluster activation was indicated.

On the other hand, recent studies of patulin synthesis suggest that several host factors are involved in the inhibition of mycotoxin accumulation in apples. Patulin accumulation during colonization of apples by *Penicillium expansum* was inhibited by the addition of extracts of the mushroom *Lentinula edodes* and of the antioxidants quercetin and umbelliferone [115,116] suggesting that metabolites—produced by the host or other organisms—can alter patulin synthesis in vivo.

We envision two possible mechanisms that might modulate fungal mycotoxin cluster activation and the accumulation of mycotoxins: (1) pathogen-modified nutritional factors present in the host that become precursors for activation of the mechanism of mycotoxin production; and (2) natural chemical factors present in the host that directly modulate the transcription factors that induce mycotoxin production.

*Pathogen-modified factors present in the host—*Accumulation of patulin by *P. expansum* was found to be strongly dependent on the secretion of D-gluconic acid (GLA) resulting from the oxidation of host sugars by the pathogen [117]. While the acidification of host tissue contributes to the activation of pathogenicity factors and colonization, it was also found that GLA accumulation may contribute both to pH reduction of the host tissue and as a precursor for patulin [118].

Downregulation of glucose oxidase (*gox2*) in *P. expansum* was accompanied by impairment in its ability to produce GLA, patulin accumulation and apple colonization. Using Δ*gox2* mutants, it was observed that the higher the impairment in GLA accumulation, the higher the inhibition of relative expression of the patulin BGC gene *idh* and patulin accumulation [118,119].

Ammonia produced by the pathogen and detected on the edge of *P. expansum*-colonized tissues under host conditions of lower sucrose level led to enhanced patulin accumulation [120]. This type of response is probably the result of reduced free sucrose levels at the leading edge of the colonized tissue inducing amino acid metabolism, in contrast to the free sugar presence at the center of the decay, where macerated tissue provides a sufficient carbon source [120]. This host effect was confirmed by direct NH_4_Cl treatments to *P. expansum* colonizing fruits, where ammonia treatment induced patulin accumulation in the colonized tissue. Interestingly, ammonia induced patulin accumulation concurrently with transcript activation of *pacC* and patulin BCG genes, indicating the regulatory effect of ammonia on *pacC* transcript expression under acidic conditions [120]. These findings indicate not only that external factors affect fungal growth, but also that intrinsic metabolic changes lead to different levels of sugar availability occurring in the fruit during ripening and pathogenesis, which may affect first and secondary fungal metabolism and mycotoxin accumulation.

Another pathogen not present in fruits, for which sugar impacted toxin production, is *Stagonospora nodorum*. Deletion of the transcription factor gene *SnStuA* played a key role in regulating central C metabolism, with glycolysis, the tricarboxylic acid cycle, and amino acid synthesis in the mutants positively regulating the synthesis of the mycotoxin alternariol [121]. These data suggest that the metabolism of fermentable C sources negatively affects mycotoxin production in some cases. This study also uncovered a multitude of regulatory targets of fungal genes in plants, suggesting the possibility that other fungal–host interactions affect mycotoxin accumulation.

*Natural host factors affecting the pathogen—*Efforts to identify host factors that are potentially involved in patulin regulation have centered on the role of sucrose in impacting patulin levels. Kumar et al. [117] demonstrated that sucrose, the main sugar usually present in fruit, modulates patulin accumulation in a dose-responsive pattern by directly regulating the expression of the global regulator of secondary metabolism, *laeA*. An increase in sucrose culture amendment from 15 to 175 mM decreased both patulin accumulation and *laeA* expression by 175- and 5-fold, respectively. These results may tie into the ammonia levels, since the highest patulin accumulation was observed in the presence of low sucrose level, conditions that induce ammonia accumulation by the pathogen [122]. This suggests that limiting sugar levels, probably resulting from intact cells in unripe fruit, may be a mechanism for activating mycotoxin synthesis that differs from that in cell walls from mature ripe cells, which activates different processes of fungal metabolism. Interestingly, negative regulation of *creA* compared to *laeA* was observed at different sucrose levels, also indicating the importance of sugar regulation in these transcription factors. These data support the view that host nutritional factors, as a result of fruit maturity, may differentially contribute to regulation of the patulin BGC.

Using freshly harvested fruit sampled at increasing stages of maturity, the ability of *P. expansum* to colonize fruit and accumulate patulin was analyzed in apple fruit at increasing maturity. The wild-type strain of *P. expansum* (WT) showed increasing patulin accumulation, from 0.2 to 1.5 mg g^−1^ fresh weight as the fruit matured, and the total soluble solids of the fruits (TSS) increased from 12.5% and 13.5% to 13.96% in the first, second, and third harvests, respectively. Thus the WT Pe-21 strain showed an increasing trend for patulin accumulation in apples in progressive harvests with progressive aggressiveness of the pathogen. The differential levels of sugar, found both in vitro and in vivo, suggest that internal metabolic factors may further contribute to the regulation of the metabolic cluster and patulin accumulation.

## 4. Conclusions

Changes in fruit maturity and ripening are accompanied by significant nutritional factors that determine the quality of the fruit. While many reports indicate that mycotoxin accumulation is dependent on a variety of environmental factors [5,123], there are few examples showing how changes in natural host metabolites during ripening affect fungal metabolites and the global regulation of mycotoxin synthesis. This is important given the differential levels of mycotoxin accumulation during maturation and ripening. Interestingly, until now, there have been few reports indicating the importance of changes in the level of organic acids and other natural fruit compounds, such as phenols. In *Aspergillus*, the phenolic antioxidants gallic acid, vanillic acid, protocatechuic acid, 4-hydroxybenzoic acid, catechin, caffeic acid, and chlorogenic acid, which are present in fruit, were studied for their effects on OTA production. Whereas *Aspergillus ochraceus* was not inhibited by any of these compound, the effects of each compound on OTA production were variable, suggesting that species-specific OTA production and response to phenolic compounds may be influenced by different and as yet unreported mechanisms affecting OTA accumulation [120].

A similar approach should be taken for organic acids, such as malic and citric acids, which are present in ripening fruit. A recent report indicates that organic acids may have antifungal activity against mycotoxigenic pathogens and an inhibitory effect on aflatoxin B1 accumulation [124]. If we take into account the dynamic changes in those natural fruit compounds and possible synergistic effects, natural host factors may regulate mycotoxin production by affecting its biosynthetic activation. Future research should focus on understanding how these natural host factors impact BGC expression and mycotoxin production during fruit colonization.
